# Mild Illness during Outbreak of Shiga Toxin−Producing *Escherichia coli* O157 Infections Associated with Agricultural Show, Australia

**DOI:** 10.3201/eid2310.161836

**Published:** 2017-10

**Authors:** Bhakti R. Vasant, Russell J. Stafford, Amy V. Jennison, Sonya M. Bennett, Robert J. Bell, Christine J. Doyle, Jeannette R. Young, Susan A. Vlack, Paul Titmus, Debra El Saadi, Kari A.J. Jarvinen, Patricia Coward, Janine Barrett, Megan Staples, Rikki M.A. Graham, Helen V. Smith, Stephen B. Lambert

**Affiliations:** Queensland Health, Brisbane, Queensland, Australia (B.R. Vasant, R.J. Stafford, A.V. Jennison, S.M. Bennett, R.J. Bell, C.J. Doyle, J.R. Young, S.A. Vlack, P. Titmus, D. El Saadi, K.A.J. Jarvinen, M. Staples, R.M.A. Graham, H.V. Smith, S.B. Lambert);; University of Queensland, Brisbane (S.A. Vlack, S.B. Lambert);; Queensland Treasury, Brisbane (P. Coward);; Department of Agriculture and Fisheries, Brisbane (J. Barrett)

**Keywords:** Escherichia coli, bacteria, Shiga toxin−producing E. coli, STEC, Shiga toxin, illness, infections, outbreak, hemolytic uremic syndrome, HUS, agricultural show, whole-genome sequencing, Brisbane, Australia

## Abstract

During a large outbreak of Shiga toxin−producing *Escherichia coli* illness associated with an agricultural show in Australia, we used whole-genome sequencing to detect an IS*1203*v insertion in the Shiga toxin 2c subunit A gene of Shiga toxin−producing *E. coli*. Our study showed that clinical illness was mild, and hemolytic uremic syndrome was not detected.

Shiga toxin−producing *Escherichia coli* (STEC) is a major cause of serious human gastrointestinal illness and has the potential to cause life-threatening complications, such as hemolytic uremic syndrome (HUS) (*1*). An average of 0.4 cases of STEC illness per 100,000 persons per year are reported to public health authorities in Australia (*2*). Disease severity can range from asymptomatic infection to serious and sometimes fatal illness, particularly in young children and the elderly (*3*,*4*).

Healthy ruminants, particularly cattle, are the reservoir for STEC (*5*). Human infection with STEC usually occurs as a result of inadvertent ingestion of fecal matter after consumption of contaminated food, water, or unpasteurized milk; contact with animals or their environments; or secondarily, through contact with infected humans (*4*,*5*). In the largest previously reported outbreak of STEC illness in Australia in 1995, which was associated with consumption of mettwurst (uncooked, semidry, fermented sausages), HUS developed in 23 of the 51 case-patients identified, and there was 1 death (*6*).

## The Study

A multidisciplinary incident management team was established to investigate an outbreak of STEC illness associated with an annual agricultural show in Brisbane, Queensland, Australia, in August 2013 (Technical Appendix). The incident management team defined primary and secondary outbreak cases (Technical Appendix). Persons with laboratory-confirmed STEC infection associated with the outbreak and their household contacts were followed up until the point of microbiological evidence of clearance, which was defined as 2 consecutive negative stool samples collected >24 h apart (*7*).

Case-patients and contacts with a high risk for transmission (persons <5 years of age; persons unable to maintain good hygiene; or childcare, healthcare, aged care, or food preparation workers) were advised to avoid childcare and work settings in accordance with Queensland Health Guidelines (*7*). Enhanced surveillance measures were implemented to assist with case finding (Technical Appendix). Medical practitioners were requested to avoid use of antimicrobial drugs for suspected case-patients with STEC infections because of previously reported associations between antimicrobial drug use and HUS (Technical Appendix).

We developed a case−control study to obtain additional information related to animal contact, hand hygiene, and food consumption at the agricultural show (Technical Appendix). We analyzed data by using Epi Info 7 (Centers for Disease Control and Prevention, Atlanta, GA, USA) (Technical Appendix).

STEC identified from human, environmental, and animal samples were serotyped for O and H antigens (Technical Appendix). Expression of Shiga toxin 1 (*stx1*) and *stx2* genes was determined for selected isolates (Technical Appendix). Shiga toxin gene subtyping and whole-genome sequencing (WGS) analysis was performed (Technical Appendix).

**Figure 1:**
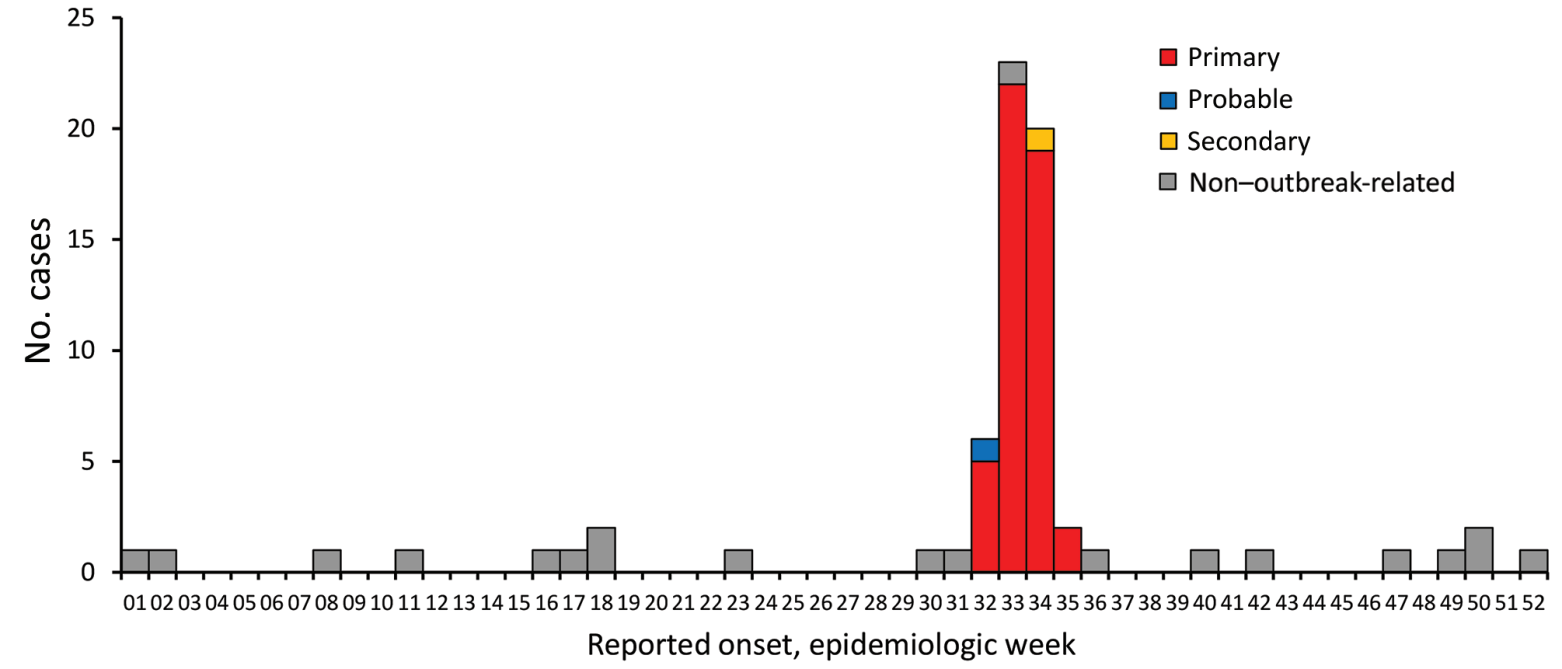
Illness onset dates for persons with cases of Shiga toxin−producing *Escherichia coli* illness associated with an agricultural show and non–outbreak-related cases, Brisbane, Queensland, Australia, 2013. Onset dates for 6 primary cases were not available. There was 1 asymptomatic secondary case.

During August 21−September 27, 2013, we identified 57 outbreak-associated laboratory-confirmed case-patients with STEC infection: 54 confirmed primary case-patients, 1 probable primary case-patient, and 2 secondary case-patients (Figure 1). Of the 57 case-patients, 32 (56%) were female. Case-patients ranged in age from 1 to 77 (median 9) years; 31 (56%) case-patients were <10 years of age. Median incubation period after attending the agricultural show was 4 (range 1–11, 25th–75th percentile 3–5) days.

Case-patients reported diarrhea (96%), abdominal pain (93%), bloody diarrhea (41%), and fever (32%) (Table 1). Seven case-patients were hospitalized. No cases of HUS or deaths were reported.

**Table 1:** Frequency of symptoms among persons, by age group, with cases of Shiga toxin−producing *Escherichia coli* illness associated with an agricultural show, Brisbane, Queensland, Australia, 2013

Symptom (self reported)	Age group, y. no. positive/no. responded (%)			
	All	1−4	5−14	>15
Diarrhea	48/50 (96)	16/17 (94)	15/15 (100)	17/18 (94)
Bloody diarrhea*	19/46 (41)	1/16 (6)	7/13 (54)	11/17 (65)
Abdominal pain	37/40 (93)	12/14 (86)	9/9 (100)	16/17 (94)
Fever	14/44 (32)	6/16 (38)	4/14 (29)	4/14 (29)

*Older children and adults were significantly more likely to report bloody diarrhea than children <5 years of age (p = 0.001 by χ^2^ test for linear trend).

Public Health Units followed up 40 case-patients until microbiological evidence of clearance; the remaining case-patients were lost to follow-up. Median duration of STEC excretion among primary case-patients was 18 (range 2−52) days (Table 2). After 27 days and 6 recurrent stools positive for STEC, and after acute diarrheal illness had resolved, 1 child was given azithromycin on day 40 for 3 days to hasten decolonization. Two consecutive stool specimens obtained >48 h after treatment with antimicrobial drugs was stopped were negative for STEC in this child. This patient did not have any adverse effects from azithromycin treatment.

**Table 2:** Clearance of STEC from stool samples of persons, by age group, during outbreak of Shiga toxin−producing *Escherichia coli* illness associated with an agricultural show, Brisbane, Queensland, Australia, 2013*

Characteristic	All, n = 40	1−4 y, n = 12	5−14 y, n = 13	>15 y, n = 15
Median clearance, d (range)	18 (2–52)	29 (5–37)	23 (2–52)	12 (2–28)
Mean clearance, d (SD)	19 (12)	24 (9)	24 (12)	12 (9)

*Time to clearance was based on onset of diarrhea. STEC, Shiga toxin−producing *Escherichia coli.*

Forty-four of 55 primary case-patients and 28 household contacts who attended the agricultural show were included in the case−control study. Median age of case-patients was 8 (range 1−77) years, and median age of controls was 38 (range 1−70) years.

We showed by using univariate analysis that case-patients were not more likely than controls to have entered the animal nursery at the show., Case-patients were more likely than controls to have had contact with lambs or goats, fed the animals, or had their hands licked by animals (Technical Appendix).

**Figure 2:**
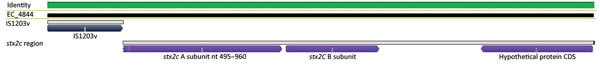
Alignment of genomic region from a representative isolate (EC_4844) showing insertion of IS*1203*v in the Shiga toxin 2 (*stx2*) gene region of Shiga toxin−producing *Escherichia coli* associated with an agricultural show, Brisbane, Queensland, Australia, 2013. CDS, coding DNA sequence.

We identified the same multilocus variable number tandem repeat and *stx* subtype genotype of *E. coli* O157:H- in human case-patients, animal bedding from the animal nursery before disposal, and fecal samples collected from lambs, goats, and calves (Technical Appendix). WGS and read mapping to an *E. coli* O157 reference genome showed that of the human, animal, and environmental isolates analyzed, all contained an IS*1203*v insertion that resulted in deletion of the first 494 bp of the *stx2c* subunit A gene (Figure 2). Expression of *stx2* was not detected in these isolates by Immunocard STAT! EHEC (Meridian Bioscience, Cincinnati, OH, USA) and Shiga toxin Quik Chek (Alere, Waltham, MA, USA) lateral flow devices. No additional *stx2* genes were identified, and no disruptions were detected in the *stx1* gene regions of any of the isolates.

## Conclusions

We found that STEC infection was associated with feeding lambs or goats, feeding animals, and having the hands licked by animals. The course of *E. coli* O157 infection was relatively mild; no cases of HUS were associated with this outbreak. Heiman et al. found that of 4,928 cases of 390 *E. coli* O157 illness outbreaks in the United States during 2003–2012, HUS was detected in 299 cases (6% of illnesses) (*8*). HUS cases with *stx1+*/*stx2*−*E. coli* O157 isolates have been reported (*9*,*10*). We speculate that the absence of severe complications in this outbreak might have been caused, in part, by disruption of the *stx2* subunit A gene by the IS*1203*v insertion, which resulted in lack of expression or a nonfunctional Stx2 toxin.

The proportion of case-patients reporting bloody diarrhea (19/46, 41%) was also lower than previously reported. Ethelberg et al. reported that 69% (56/81) of case-patients in Denmark infected with *E. coli* O157 had bloody diarrhea (*11*). A recent retrospective cohort study from England reported that 61% (2,027/3,323) of symptomatic case-patients infected with *E. coli* O157 had bloody diarrhea. Bloody diarrhea was reported to be a risk factor for HUS (odds ratio 2.10; p = 0.001) (*12*). In the outbreak we studied, children <5 years of age were less likely than older children and adults to report bloody diarrhea. STEC infection should be actively considered for young children with nonbloody diarrhea who were exposed to potential sources of STEC.

In this outbreak, 1 child was given azithromycin for 3 days to hasten decolonization some weeks after the acute diarrheal illness had resolved. Antimicrobial drugs are generally not recommended to hasten STEC decolonization because of major associations with HUS (*13*). Recommendations to avoid high-risk activities (such as childcare attendance) might place a major socioeconomic burden on STEC carriers and their families. Further studies are required to assess whether WGS can provide useful information for safe administration of antimicrobial drugs for treatment of acute illness caused by STEC, or when chronic shedding becomes established.

Our comprehensive study of a large outbreak *E. coli* O157 illness, characterized by an IS*1203*v insertion disrupting the *stx2c* subunit A gene, showed mild clinical illness and an absence of HUS. Further characterization by virulence studies on isolates with this *stx2c* subunit A gene disruption might provide further insights into the mild illness caused by this strain.

Technical Appendix Additional information on mild illness during outbreak of Shiga toxin−producing *Escherichia coli* O157 infections associated with agricultural show, Australia.
