# Supplementary material for: Mild Illness during Outbreak of Shiga Toxin−Producing Escherichia coli O157 Infections Associated with Agricultural Show, Australia
Source: Emerg Infect Dis. 2017 Oct;23(10):1686–9. doi: 10.3201/eid2310.161836 (PMC5621556; doi:10.3201/eid2310.161836)
Supplement: Technical Appendix — Additional information on mild illness during outbreak of Shiga toxin−producing Escherichia coli O157 infections associated with agricultural show, Australia. [file 16-1836-Techapp-s1.pdf]

Article DOI: http://doi.org/10.3201/eid2310.161836

Mild Illness during Outbreak of Shiga Toxin–Producing *Escherichia coli* O157 Infections Associated with Agricultural Show, Australia

Technical Appendix

Outbreak

On August 21, 2013, Queensland Health Forensic and Scientific Services (QHFSS), the public health reference laboratory in Coopers Plains, Queensland, Australia, informed public health authorities of 2 case-patients with Shiga toxin–producing *Escherichia coli* O157 (STEC) illness in Brisbane. The next day, 2 additional case-patients were reported. All 4 case-patients attended the annual agricultural show in Brisbane, the capital of Queensland, a state of Australia, and had no other exposures in common. The agricultural show was open to the public during August 8–17. The show provided opportunities for visitors to see and pet farm animals. More than 400,000 persons visited the show in 2013.

On August 23, after identification of the common exposure, the Queensland Communicable Diseases Unit convened an incident management team consisting of public health units, QHFSS, and OzFoodNet (a national network that investigates foodborne disease outbreaks) (*1*). Biosecurity Queensland (the Queensland Government agency that leads efforts to prevent, respond to, and recover from pests and diseases threatening agricultural prosperity, the environment, social amenity and human health) and Workplace Health and Safety Queensland (a government agency that enforces workplace health and safety laws) provided interagency support.

Enhanced Surveillance

On August 23, the Queensland Communicable Diseases Unit alerted all pathology laboratories, hospital emergency departments, infectious diseases physicians, and general practitioners in the Brisbane metropolitan area about the outbreak. Medical practitioners were

requested to submit bloody stool specimens for STEC testing and to avoid use of antimicrobial drugs for potential cases because of previously reported associations between antimicrobial drug use and hemolytic uremic syndrome (HUS) (*2*). Pathology laboratories were requested to review test results for recently collected bloody stool specimens and forward these specimens to QHFSS for STEC testing. Case finding was assisted by a media release on August 23 that alerted the public about cases of STEC associated with the agricultural show and advised persons who attended the show and in whom bloody, severe, or persistent diarrhea subsequently developed to seek medical attention (*3*).

Case Definition

A confirmed primary case was defined as a case in a person in whom all 4 virulence genes (Shiga toxin [*stx*], intimin [*eaeA*], enterohemolysin [*ehxA*], and autoagglutinating adhesion [*saa*]) were detected by PCR in a stool specimen and who had visited the annual agricultural show and whose onset of illness or whose stool specimen collection date was within 14 days of attendance. A probable primary case was similarly defined, except for differences in the STEC strain and virulence genes. A secondary case was also similarly defined, except that the case was epidemiologically linked to a primary case, and that the secondary case-patient did not attend the agricultural show or the onset of illness was >14 days after attending the agricultural show. The case definition excluded case of STEC illness that were not associated with the agricultural show. The end of the outbreak was defined by an absence of new cases during 2 incubation periods (28 days) of the onset of illness in the last confirmed outbreak-associated case of STEC illness.

HUS was defined as the presence of microangiopathic hemolytic anemia, thrombocytopenia (i.e., platelet count <150,000/μL), and renal insufficiency. Renal insufficiency was defined as a creatinine level greater than the upper limit of the reference range for age (*4*).

Case Investigations and Case–Control Study

Public health units administered the standard Queensland Health STEC case questionnaire to all case-patients identified in this outbreak (*5*). The proportion of case-patients who reported symptoms was based on persons for whom data for the specific field was available. For the case–control study, a supplementary questionnaire was developed to obtain additional

information related to animal contact, hand hygiene, and food consumption at the agricultural show.

The supplementary questionnaire was administered to primary case-patients. Controls for the case–control study were asymptomatic household contacts who visited the agricultural show and had negative test results for STEC infection. The parent or guardian were interviewed for case-patients and household contacts ≤14 years of age. For persons 15–17 years of age, verbal consent of the parent or guardian was obtained before the interview was conducted.

Statistical Analysis

Data were analyzed by using Epi Info 7 (Centers for Disease Control and Prevention, Atlanta, GA, USA). Univariate analysis generating crude odds ratios with 95% CIs was used to investigate associations between potential risk factors and STEC infection (Technical Appendix Table 1). Variables with a p value ≤0.05 were included in multivariable logistic regression models for further assessment. Potential collinear variables were assessed by using the Cramer V statistic (Stata version 11.0; StataCorp LLC, College Station, TX, USA). To assess the effect of using other household members as controls, we also performed matched analyses adjusting for age and sex.

Environmental Investigation

Environmental health officers reviewed infection prevention and control measures at the agricultural show animal pavilion, including handwashing facilities, signs encouraging visitors to wash their hands, and animal waste disposal. Environmental samples (including remaining composite straw, shavings, and visible manure) were obtained from the animal nursery for laboratory testing after the show had ended. Biosecurity Queensland coordinated a risk assessment of the animal contact areas, traced animals that had been on display, and subsequently collected animal fecal samples for STEC testing.

Laboratory Investigation

STEC Detection

All specimens were inoculated into *E. coli* enrichment broth (Difco, Franklin Lakes, NJ, USA) for 16–24 h at 37°C, which was then plated onto MacConkey agar for 16–24 h at 37°C. Resulting growth was screened for the *stx1*, *stx2*, *eaeA*, *ehxA*, and *saa* genes (*6*). Cultures positive for *stx1* and/or *stx2* were subcultured to isolate pure growth for further testing. The

expression of *stx1* and *stx2* was determined on selected isolates by using Immunocard STAT! EHEC (Meridian Bioscience, Cincinnati, OH, USA) and Shiga toxin Quik Chek (Alere, Waltham, MA, USA) tests according to the manufacturer's instructions. All *stx1* and/or *stx2* gene–positive *E.coli* isolates were serotyped for O and H antigens (H typing performed by the Microbiological Diagnostic Unit, Melbourne, Victoria, Australia).

Molecular Characterization

Shiga toxin gene subtyping for *stx1* and *stx2* was performed for all isolates available (*7*–*10*). Multilocus variable number tandem repeats analysis (MLVA) was also performed by using 2 schemes, 1 specific for all *E. coli* isolates and 1 specific for to serogroup O157 isolates (*11*,*12*).

Whole-genome sequencing was performed for selected isolates and demonstrated the molecular profile associated with the outbreak (3 human isolates, 1 ovine isolate, 1 caprine isolate, 1 bovine isolate, 1 bedding isolate). A total of 300 ng of genomic DNA was sheared by ultrasonification to 300-bp fragments by using an S220/E220 ultrasonicator (Covaris, Woburn, MA, USA).

Samples were prepared into barcoded fragment libraries by using the Ion Plus Fragment Library Kit and IonXpress barcode adaptors, and sequenced on an Ion Torrent PGM by using the Ion PGM Hi-Q Sequencing Kit, the Ion PGM Hi-Q Chef Kit, and 316v2 chips (Life Technologies, Waltham, MA, USA) according to the manufacturer's instructions. Libraries were assessed by using TapeStation 2200 with High Sensitivity D1000 Screen Tape (Agilent Technologies, Santa Clara, CA, USA). Quality check filtering, trimming, and adaptor sequence removal was performed by Torrent Suite software (Life Technologies, Waltham, MA). Raw reads are located in the sequence read archive under BioProject PRJNA342737.

FASTQ sequences were mapped to the reference genome of *E. coli* O157:H7 strain Sakai (NC_002695) by using Geneious R7 (http://www.geneious.com/). De novo assemblies produced by the Geneious R7 assembler were used to identify in silico multilocus sequence typing alleles in Ridom SeqSphere+ according to a standard scheme (http://mlst.warwick.ac.uk/mlst/dbs/Ecoli) (*13*).

Ethics

Ethical approval was not required because all activities contributed to the public health response in identifying, characterizing, and controlling disease. Outbreak prevention and control measures are covered under the Public Health Act 2005, Queensland (*14*).

Results

Environmental Investigation

The 2013 agricultural show displayed >10,000 animals and included sections where direct contact between visitors and animals could occur. The animal boulevard included a large animal nursery where visitors could pat and feed farm animals, including goats, lambs, calves, piglets, chicks, ducklings, donkeys, and turkeys. A milking demonstration took place in an area adjacent to the animal nursery and visitors were invited to milk a cow. Unpasteurized milk was not served. Visitors could also view the birth of lambs that took place in an enclosed booth. The birthed lambs were available for supervised petting after ≥24 h after veterinary clearance. Other animals displayed in the animal boulevard and other pavilions were less accessible to the public for direct contact.

The number of visitors in the animal nursery was not restricted. Limited unsupervised handwashing facilities were available opposite the exit of the animal nursery. Hand sanitizers were available in other areas. Signs in animal contact areas encouraged visitors to wash their hands. Staff at the agricultural show regularly removed animal waste from animal contact areas.

Laboratory Investigation

Stool samples from 56 of 57 case-patients showed identical virulence gene profiles, consisting of *stx1*, *stx2*, *eaeA*, and *ehxA* . The virulence gene profile of the remaining probable primary case-patient was only *stx2* and *ehxA*. Twenty bovine, 4 ovine, and 2 caprine fecal samples were tested from animals traced to other properties after the show had ended. Serotype O157:H− was confirmed from 51 of the human cases, and also from ovine, caprine, and bovine feces, and the animal bedding sample. All O157:H− isolated from animal and environmental sources displayed the same MLVA profiles (6_8_2_9_4_7_8_2_3_8 and 11–7-13–4-5–6-4–9) (Technical Appendix Table 2), *stx1a* and *stx2c* subtypes, and sequence type ST11, and 2/51 of human isolates differed by 1 allele in 1 of the MLVA profiles. Although *E. coli* O157 has

frequently been reported to belong to sequence type 11 (*13*), the MLVA profiles were novel to the Queensland collection of previously typed STEC isolates (n = 112).

References

1. OzFoodNet. A network to enhance the surveillance of foodborne diseases in Australia. Commonwealth of Australia 2005 [cited 2016 Sep 25]. http://www.ozfoodnet.gov.au/internet/ozfoodnet/publishing.nsf/Content/copyright-1

2. Wong CS, Jelacic S, Habeeb RL, Watkins SL, Tarr PI. The risk of the hemolytic-uremic syndrome after antibiotic treatment of *Escherichia coli* O157:H7 infections. N Engl J Med. 2000;342:1930–6. PubMed http://dx.doi.org/10.1056/NEJM200006293422601

3. Queensland Health. Queensland Health issues public health alert for STEC. Queensland Health August 22, 21013 [cited 2016 Sep 25]. https://www.facebook.com/QLDHealth/posts/460032644104823

4. Freedman SB, Xie J, Neufeld MS, Hamilton WL, Hartling L, Tarr PI; Alberta Provincial Pediatric Enteric Infection Team (APPETITE). Shiga toxin–producing *Escherichia coli* infection, antibiotics, and risk of developing hemolytic uremic syndrome: a meta-analysis. Clin Infect Dis. 2016;62:1251–8. PubMed http://dx.doi.org/10.1093/cid/ciw099

5. Queensland Government. EHEC/HUS case report form. Queensland Government February 4, 2003 [cited 2016 Sep]. https://www.health.qld.gov.au/foodsafety/documents/EHECCRF.pdf

6. Paton AW, Paton JC. Direct detection and characterization of Shiga toxigenic *Escherichia coli* by multiplex PCR for stx_1, stx_2, *eae, ehxA*, and *saa.* J Clin Microbiol. 2002;40:271–4. PubMed http://dx.doi.org/10.1128/JCM.40.1.271-274.2002

7. Persson S, Olsen KE, Ethelberg S, Scheutz F. Subtyping method for *Escherichia coli* Shiga toxin (verocytotoxin) 2 variants and correlations to clinical manifestations. J Clin Microbiol. 2007;45:2020–4. PubMed http://dx.doi.org/10.1128/JCM.02591-06

8. Wang G, Clark CG, Rodgers FG. Detection in *Escherichia coli* of the genes encoding the major virulence factors, the genes defining the O157:H7 serotype, and components of the type 2 Shiga toxin family by multiplex PCR. J Clin Microbiol. 2002;40:3613–9. PubMed http://dx.doi.org/10.1128/JCM.40.10.3613-3619.2002

9. Osek J. Development of a multiplex PCR approach for the identification of Shiga toxin–producing *Escherichia coli* strains and their major virulence factor genes. J Appl Microbiol. 2003;95:1217–25. PubMed http://dx.doi.org/10.1046/j.1365-2672.2003.02091.x

10. Beutin L, Miko A, Krause G, Pries K, Haby S, Steege K, et al. Identification of human pathogenic strains of Shiga toxin–producing *Escherichia coli* from food by a combination of serotyping and molecular typing of Shiga toxin genes. Appl Environ Microbiol. 2007;73:4769–75. PubMed http://dx.doi.org/10.1128/AEM.00873-07

11. Løbersli I, Haugum K, Lindstedt BA. Rapid and high resolution genotyping of all *Escherichia coli* serotypes using 10 genomic repeat-containing loci. J Microbiol Methods. 2012;88:134–9. PubMed http://dx.doi.org/10.1016/j.mimet.2011.11.003

12. Hyytia-Trees E, Lafon P, Vauterin P, Ribot EM. Multilaboratory validation study of standardized multiple-locus variable-number tandem repeat analysis protocol for Shiga toxin–producing *Escherichia coli* O157: a novel approach to normalize fragment size data between capillary electrophoresis platforms. Foodborne Pathog Dis. 2010;7:129–36. PubMed http://dx.doi.org/10.1089/fpd.2009.0371

13. Wirth T, Falush D, Lan R, Colles F, Mensa P, Wieler LH, et al. Sex and virulence in *Escherichia coli:* an evolutionary perspective. Mol Microbiol. 2006;60:1136–51. PubMed http://dx.doi.org/10.1111/j.1365-2958.2006.05172.x

14. The State of Queensland. Public Health Act 2005. The State of Queensland 2005 [cited 2016 Sep 26]. https://www.legislation.qld.gov.au/LEGISLTN/CURRENT/P/PubHealA05.pdf

Technical Appendix Table 1. Univariate analysis of selected animal and environmental exposures for *Escherichia coli* O157:H– isolates obtained during outbreak associated with agricultural show, Australia, 2013*

	Proportion exposed, no. positive/no. tested (%)			
Exposure	Cases-patients	Controls	Unadjusted odds ratio (95% CI)	p value
Interaction with animals				
Attend animal boulevard	42/44 (95.5)	24/28 (85.7)	3.5 (0.6–20.5)	0.22
Attend animal nursery	39/43 (90.7)	23/26 (88.5)	1.3 (0.3–6.2)	1.00
Pet/touch lambs	32/37 (86.5)	13/22 (59.1)	4.4 (1.2–15.8)	0.03†
Pet/touch calves	30/38 (79.0)	13/22 (59.1)	2.6 (0.8–8.2)	0.10
Pet/touch piglets	4/31 (12.9)	2/18 (11.1)	1.2 (0.2–7.2)	1.00
Pet/touch goats	30/36 (83.3)	9/18 (50.0)	5.0 (1.4–17.9)	0.02†
Pet/touch chicks	9/37 (24.3)	2/18 (11.1)	2.6 (0.5–13.4)	0.31
Pet/touch ducklings	5/37 (13.5)	2/18 (11.1)	1.3 (0.2–7.2)	1.00
Pet/touch donkeys	9/35 (25.7)	3/18 (16.7)	1.7 (0.4–7.4)	0.73
Pet/touch turkeys	1/37 (2.7)	1/17 (5.9)	0.4 (0.0–7.6)	0.53
Pet/touch llama/alpacas	6/34 (17.7)	3/19 (15.8)	1.1 (0.3–5.2)	1.00
Pet/touch cattle at open stalls	14/41 (34.2)	8/27 (29.6)	1.2 (0.4–3.5)	0.70
Contact with animal manure	10/26 (38.5)	5/19 (26.3)	1.8 (0.5–6.4)	0.53
Attend milking barn	4/42 (9.5)	0/27 (0.0)	ND	0.15
Attend little miracles‡	14/42 (33.3)	7/26 (26.9)	1.4 (0.5–4.0)	0.79
Pet/touched newborn lamb	1/14 (7.1)	0/7 (0.0)	ND	1.00
Fed animals by hand	27/37 (73.0)	5/21 (23.8)	8.6 (2.5–29.8)	<0.001
Animals licked hands	23/33 (69.7)	4/20 (20.0)	9.2 (2.4–34.6)	<0.001†§
Hygiene				
Washed hands upon exiting	37/38 (97.4)	18/21 (85.7)	6.2 (0.6– 63.5)	0.13
Used running water only	1/44 (2.3)	0/28 (0.0)	ND	1.00
Used soap and running water only	29/38 (76.3)	14/20 (70.0)	1.4 (0.4–4.6)	0.75
Used running water and hand gel	1/43 (2.3)	2/28 (7.1)	0.3 (0.0–3.5)	0.56
Used hand gel only	6/44 (13/6)	2/28 (7.1)	2.1 (0.4–1.0)	0.47
Selected food exposures				
Dagwood dog¶	15/40 (37.5)	10/26 (38.5)	1.0 (0.3–2.7)	0.94
German sausage	5/40 (12.5)	4/26 (15.4)	0.8 (0.2–3.2)	0.73
Strawberry sundae	23/43 (53.5)	12/27 (44.4)	1.4 (0.5–3.8)	0.46
Italian meat balls	0 (0.0)	0 (0.0)	ND	NA
Beef burger	0 (0.0)	0 (0.0)	ND	NA
Steak sandwich	0 (0.0)	0 (0.0)	ND	NA

*NA, not applicable; ND, not defined because of no persons in the case-patients or control groups.
†These associations remained significantly different when analysis was restricted to children <18 y of age.
‡An audience observed the birth of lambs within an enclosed booth. After 24 h and clearance by a veterinarian, the lambs were available for petting.
§In the final unmatched multivariable model, having hands licked by animals (adjusted odds ratio [OR] 11.7, 95% CI 2.4–58.4) was found to be associated with infection after adjusting for all other variables in the model, including age and sex. Matched analyses using conditional logistic regression produced a similar estimate of effect (adjusted OR 12.5, 95% CI 0.95–162.9), although not quite reaching significance for having hands licked by an animal.
¶A hotdog sausage deep-fried in batter and served on a stick.

Technical Appendix Table 2. Molecular typing of environmental, animal, and patient *Escherichia coli* O157:H– isolates obtained during outbreak associated with agricultural show, Australia, 2013*

Source of isolate	*stx* gene subtype	*E. coli* generic MLVA profile	O157 MLVA profile	MLST†
Patients (49/51 from whom isolates were identified)	*stx1a, stx2c*	6_8_2_9_4_7_8_2_3_8	11–7–13–4-5–6-4–9	ST11
Ovine feces	*stx1a, stx2c*	6_8_2_9_4_7_8_2_3_8	11–7–13–4-5–6-4–9	ST11
Bovine feces	*stx1a, stx2c*	6_8_2_9_4_7_8_2_3_8	11–7–13–4-5–6-4–9	ST11
Caprine feces	*stx1a, stx2c*	6_8_2_9_4_7_8_2_3_8	11–7–13–4-5–6-4–9	ST11
Animal nursery bedding	*stx1a, stx2c*	6_8_2_9_4_7_8_2_3_8	11–7–13–4-5–6-4–9	ST11

*MLST, multilocus sequence typing; MLVA, multilocus variable number tandem repeats analysis; ST, sequence type; *stx*, Shiga toxin gene.
†Extrapolated from sequencing data obtained from representative isolates for each environmental, animal, and patient group.
